# Osteo-Sarcoma of Lower Jaw

**Published:** 1876-02

**Authors:** C. S. Briggs


### ARTICLE VII.

### *Osteo-Sarcoma of Lower Jaw.*

REPORTED BY C. S. BRIGGS, A. M., M. D.

Gentlemen—The first patient I present to you this morning is W——, colored, aged 52, a small farmer and preacher, who comes from our county of Putnam for advice and treatment. He has always enjoyed perfect freedom from disease until within the last three years. About that time he discovered a small nodule on the inner part of the right side of the lower jaw, which was hard, immovable, not tender to the touch, nor painful. It has grown rapidly, until now, as you see, it is as large as the closed hand; it is of an oblong shape, and occupies both sides of the jaw bone. It is irregularly nodulated, elastic in some parts, in others very inelastic; not tender upon pressure. On the outside the skin is intact, and apparently but little diseased; but on the inner side of the jaw ulceration had set in at several points, and fungus granulations have sprouted up, accompanied with an unpleasant, ichorous discharge. By passing into the central part of the tumour a small exploring needle, it encounters spiculæ of bone; so it does when passed into other parts of the mass. The patient is not much reduced, for, although spare and thin, he says he never weighed much more than he does at present. His appetite is pretty fair, and his sleep not much disturbed. His principal inconvenience is in talking—the tumour interfering very much with articulation—and he is anxious to be relieved of the diseased mass, that he may preach to his colored brethren.

From the great firmness of the mass, together with its oblong shape, and smooth or smoothly-nodulated surface, but more especially from the detection of hundreds of spiculæ of bone in its substance, I conclude that it is a case of osteo-sarcoma.

Osteo sarcoma may form on the outer surface or in the interior of bones. In the former case it is denominated periosteal, and in the latter central.

In the case before us, the growth seems to have taken place from the outer surface of the bone. Such tumors often attain large size; most generally they are malignant, often returning after removal, and sooner or later infecting the general system.

The only alternative, in this poor man's case, gentlemen, is the removal of the portion of the lower jaw upon which the tumor is situated. The part of the jaw to be removed extends from between the incisor and the canine tooth of the right side to the right temporo-maxillary articulation.

It gives me pleasure to tell you that the great honor of being the first surgeon to attempt the amputation of the lower jaw belongs to an American, and a native of our own good State, Dr. W. H. Deaderick, of Athens; since then it has been often done by surgeons in all parts of the civilized world—not only parts of the jaw having been removed, but, in many instances, the removal of the entire jaw bone has been effected.

When the patient shall have been placed on the table, in the recumbent position, and brought fully under the influence of ether, I shall make an incision from the zygomatic process a short distance in front of the ear down to the base of the jaw, thence around to the symphisis menti, under the lower border of the bone; then pass up, vertically, to the free border of the lip. This large flap is to be rapidly dissected off from the tumor, taking care to keep as close to it as possible, without leaving any diseased structure behind. Any large vessels that may have been opened having been attended to, the mucous membrane on the inner side of the bone is to be detached, and the bone sawn through at the point selected; then, the bone having been grasped, is drawn outward, and the soft parts detached from its inner side, not forgetting that we should keep as close to the tumour as is consistent with the removal of all diseased tissues.

In separating these parts from the tumor and bone, we avoid the point and edge of the knife as much as possible, using the handle more freely.

The body and angle of the jaw having been detached from their connection, we proceed to separate the soft parts from the coronoid process, which is not always an easy task; and then, drawing the portion of jaw outward, the knife is passed close to the bone, over the condyloid process, into the articulation, which is then freely opened, and the tumor completely severed from its attachments. This last manœuvre is a most important one, from the fact of the close and intimate relations the internal maxillary artery holds to the condyle.

All hemorrhage having been checked by appropriate measures, which you understand, the flap will be brought into position, and maintained by the silver suture, which I always use. The resulting deformity will be much less than any one would imagine.

[The operation was then performed as described; the half of the jaw upon which was seated the tumor was soon exsected. Very little hemorrhage followed, because the common carotid was thoroughly compressed during the entire operation. The flap was returned to its place, and kept in situ by six or eight sutures. Cold-water dressing was ordered, and the patient carried to his room. In two weeks he was again brought before the class, with the wound entirely healed, and with comparatively little deformity. The tumor proved to be a beautiful specimen of osteo sarcoma, a section displaying a number of bony spiculæ very slender and long, between which was a gelatino cartilaginous deposit.]—*Nashville Journal of Medicine and Surgery.*

---

